# Therapeutic Effects of *Aloe saponaria* against Ulcerative Colitis Induced by Dextran Sulfate Sodium

**DOI:** 10.3390/cimb45020096

**Published:** 2023-02-09

**Authors:** Do Yeong Kweon, Hee Jin Song, Ji Eun Kim, You Jeong Jin, Yu Jeong Roh, Ayun Seol, Ju Min Park, Eun Suk Lee, Won Sik Choi, Dae Youn Hwang

**Affiliations:** 1 Department of Bio-Industrial Machinery Engineering/Life, Industry Convergence Research Institute, College of Natural Resources and Life Science, Pusan National University, Miryang 50463, Republic of Korea; 2 Department of Biomaterials Science (BK21 FOUR Program)/Life, Industry Convergence Research Institute, Laboratory Animals Resources Center, College of Natural Resources and Life Science, Pusan National University, Miryang 50463, Republic of Korea; 3 Department of Food Science and Nutrition, College of Human Ecology, Pusan National University, Busan 46241, Republic of Korea

**Keywords:** *Aloe saponaria*, ulcerative colitis, dextran sulfate sodium, inflammation, toxicity

## Abstract

*Aloe vera* (*A. vera*) has been studied as a treatment option for ulcerative colitis (UC), but there is a lack of scientific evidence showing whether treatment with *Aloe saponaria* (*A. saponaria*) can also be beneficial. To investigate the therapeutic potential of *A. saponaria* as a treatment for UC, clinical symptoms, histopathological characteristics of the colon, inflammatory response, and toxicity were analyzed in dextran sulfate sodium (DSS)-induced UC mice after administration of aqueous extracts of *A. saponaria* (AAS) for 7 days. The total polyphenol and tannin content of AAS was 272 µg/g and 163 µg/g, respectively. AAS exhibited significant antioxidant activity. Several clinical symptoms, including body weight, colon length, and hematochezia, remarkably improved in the DSS+AAS treated group compared to the DSS+Vehicle-treated group. In addition, similar improvements were detected in the histopathological characteristics and mucin-secreting ability in the colon of DSS-induced UC mice after the administration of AAS. The levels of infiltrated inflammatory cells and cytokine expression were significantly decreased in a dose-dependent manner in the colon of the DSS+AAS-treated group. These alterations in inflammatory response were accompanied by a significant recovery of the protein kinase C/extracellular signal-regulated kinase (PKC/ERK) and phosphatidylinositol-3-kinase/serine-threonine protein kinase (PI3K/Akt) signaling pathways. However, the levels of key markers for hepatotoxicity and nephrotoxicity consistently remained between those of the DSS+AAS-treated and the No groups. Therefore, the results of the present study provide novel evidence that AAS may improve the clinical symptoms and attenuate the inflammatory response in DSS-induced UC mice and does not have any significant hepatotoxicity or nephrotoxicity.

## 1. Introduction

Ulcerative colitis (UC) is a form of inflammatory bowel disease (IBD) characterized by inflammation of the mucosa and submucosa of the colon, with superficial erosions on the colonic wall and disruption of the mucosal barrier [1,2]. Patients present with a variety of clinical symptoms, such as weight loss, diarrhea, bloody mucopurulent stools, and abdominal cramping [3,4]. Histologically, there is a decrease in the crypt density, severe distortion of the crypt architecture, an irregular structure of the mucosal surface, and heavy diffuse transmucosal inflammation seen in the colon [5,6]. The molecular mechanisms underlying UC include stimulation of the nuclear factor κB (NF-κB) signaling pathway, involving the toll-like receptor 4 (TLR4) and peroxisome proliferator-activated receptor γ (PPARγ), as well as the activation of the nucleotide-binding oligomerization domain (NOD)-like receptor family pyrin domain containing 3 (NLRP3) inflammasomes [7,8,9]. Drugs for the treatment of UC include 5-aminosalicylic acid, glucocorticoids, immunosuppressants, biologics, and Janus kinase (JAK) inhibitors, which target the pathological and molecular pathways involved in UC. These drugs can provide symptomatic relief and lead to disease remission, but they do not cure the disease completely [10].

The anti-UC effects of bioactive compounds isolated from various plants, including arctigenin (*Arctium lappa*), boswellic acid (*Boswellia serrata*), catechin (*Camellia sinensis*), curcumin (*Curcuma longa*), gymnemic acid (*Gymnema sylvestre*), and shagoal (*Zingiber officinale*), have been investigated using various UC animal models [11]. Similarly, various phytoconstituents with anti-inflammatory and antioxidant properties have also been evaluated for their potential as treatments for UC [12]. Among these, extracts of *Pingwei san* (*P. san*) and *Heritiera littoralis* (*H. littoralis*) have been shown to improve the disease activity index score (DAI), histological score, inflammatory response, and abnormal composition of the gut microbiota in UC models [13,14]. Furthermore, several *Aloe vera* (*A. vera*) products have shown therapeutic benefits in the symptomatic treatment of UC. Treatment with *A. vera* gel significantly improved the clinical activity index, inflammation, and histological alterations in animal models with acetic acid-induced UC [15,16,17]. *A. vera* gel has also been shown to reduce the histological disease activity in patients with UC at various dosages, as well as to inhibit the inflammatory mediators in human colorectal mucosal biopsies [18,19]. A formulation of ubiquinol and *A. vera* improved inflammation, mechanical impairment, and oxidative stress in dextran sulfate sodium (DSS)-induced UC in mice, while an ethanol extract of *A. vera* showed migration effects on the body weight, colon length, and concentration of inflammatory mediators in DSS- and 2,4,6-trinitrobenzene sulfonic acid (TNBS)-induced UC models [20,21]. Moreover, improvements in mucosal barrier function and inflammation were seen in the colon of DSS-induced UC mice treated with aqueous extracts of *A. vera* [22]. However, the therapeutic effects of *Aloe saponaria* (*A. saponaria*) in treating UC have not been investigated, despite *A. vera* and *A. saponaria* having similar compositions of bioactive compounds. In addition, *A. saponaria* has received a great deal of attention and recently cultivation was begun in Korea because their leaves are known to contain 65% more saponin than those of *A. vera* [23].

The current study was undertaken to investigate the therapeutic potential of *A. saponaria* (AAS) in the treatment of UC. Specifically, the effects of AAS were evaluated through analyses of key markers, including weight loss, hematochezia, colon length, histological characteristics, mucin secretion, and inflammatory response in a DSS-induced UC model.

## 2. Materials and Methods

### 2.1. Preparation of A. saponaria (AAS)

**Figure 1:**
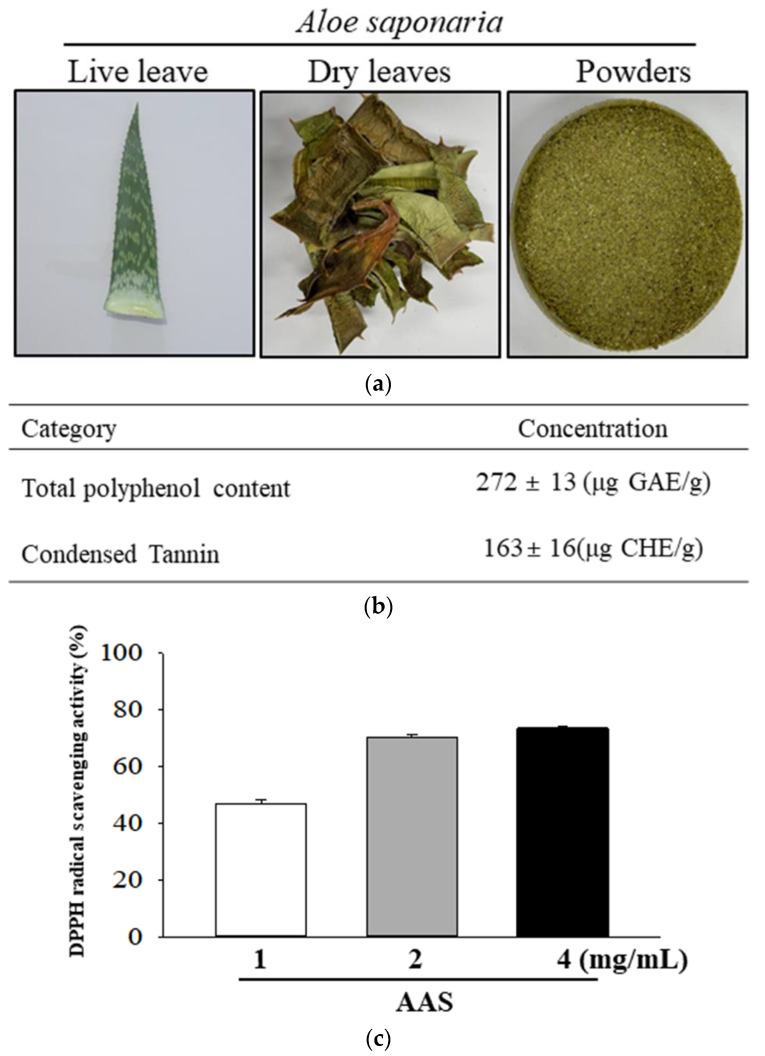
Morphology and biochemical properties of *A. saponaria.* (**a**) After drying the *A. saponaria* leaves completely, the leaves were powdered using a blender. This was used as the sample for further studies. (**b**) Total polyphenol and condensed tannin contents were determined at different concentrations of AAS. (**c**) DPPH radical scavenging activity was measured in a mixture including 0.1 mM DPPH and three concentrations of AAS (1, 2, and 4 mg/mL). These samples were assayed in duplicate by DPPH radical scavenging activity analysis. Data are reported as the mean ± SD. Abbreviations: AAS, aqueous extract of *A. saponaria*; DPPH, 1,1-diphenyl-2-picrylhydrazyl radical.

Dried leaves of AAS were provided by the Kweon Do Yeong Aloe Co. (Ulsan, Republic of Korea). The dried leaves of AAS were powdered using a pulverizer (MF-3100S, Hanil Electric Co., Seoul, Republic of Korea). The powder was filtered using a sieve with a 100 mesh (Figure 1a). Voucher specimens of AAS (WPC-22-002) were deposited at the functional materials bank of the Wellbeing RIS Center, Pusan National University. The extract solution of AAS was obtained via extraction for 4 h at 70 °C and 110 rpm, using a fixed liquor ratio (ratio of dry powdered AAS to dH_2_O solvent, 1:15) in circulating extraction equipment (SHWB-30/45; Woori Science Instrument Co., Pocheon, Republic of Korea). This solution was then passed through a 0.4 μm filter and subsequently concentrated, after which the pellets of AAS were dried at 45 °C under reduced pressure using a rotary evaporator (EYELA, Tokyo, Japan) and stored at −80 °C until use.

### 2.2. Determination of Total Polyphenol Content and Tannin

A modified Folin–Ciocalteu (F-C) assay [24] was performed to determine the total phenolic content. A 10 µL sample solution, 100 µL of distilled water, and 20 µL of F–C reagent were mixed. This mixture was left to stand for 3 min in the dark, and then 20 µL of 10% sodium nitrate and 70 µL of distilled water were added, and it was further left to stand for 1 h in the dark. The absorbance at 725 nm was measured on a microplate reader. The phenolic content was expressed as gallic acid equivalents (GAE).

The condensed tannin content was determined using colorimetric assays that partially modified the vanillin method [25]. Then, 10 mg of each extract was added to 1 mL of methanol and shaken for 20 min to obtain a supernatant. The collected supernatant was diluted for the test. Next, 100 µL of the extract was added to 500 µL of vanillin-HCl solution (1% vanillin/methanol and 8% HCl mixed 1:1). The mixture was incubated for 20 min at 30 °C, and the absorbance was read at 500 nm. The total condensed tannin content was calculated using the standard curve using a (+)-catechin hydrate. The total condensed tannin content was expressed as (+)-catechin hydrate equivalents (CHE).

### 2.3. Free Radical Scavenging Activity Analysis

The 2,2-diphenyl-1-picrylhydrazyl (DPPH) radical scavenging activity was determined using a previously described method [26,27]. Briefly, a lyophilized sample of AAS was dissolved in dimethyl sulfoxide (DMSO) (100 µL) to obtain 3 different solution concentrations of AAS (1, 2, and 4 mg/mL), which were then mixed with 100 µL of 0.1 mM DPPH (Sigma-Aldrich Co., St. Louis, MO, USA) in a 95% ethanol solution, or with 100 µL 95% ethanol solution (control), followed by incubation at 37 °C for 10 min in dark conditions. A VersaMax ^TM^ plate reader (Molecular Devices, Sunnyvale, CA, USA) was used to determine the absorbance of the reaction mixture at 540 nm. The DPPH radical scavenging activity of AAS was expressed as the percentage decrease in absorbance, relative to the control.

### 2.4. Experimental Design for the Animal Study

The protocol to examine the effects of AAS in animals with UC was reviewed and approved by the Pusan National University-Institutional Animal Care and Use Committee (PNU-IACUC) based on their ethical procedures for scientific care (Approval Number PNU-2022-0128). All adult C57BL/6 mice (8-week-old, Male) were purchased from Samtako BioKorea Inc. (Osan, Republic of Korea) and maintained at the Pusan National University-Laboratory Animal Resources Center, accredited by the Korea Food and Drug Administration (KFDA) (Accredited Unit Number: 000231) and The Association for Assessment and Accreditation of Laboratory Animal Care (AAALAC) International (Accredited Unit Number; 001525). All animals were provided *ad libitum* access to a standard irradiated chow diet (Samtako BioKorea Inc.) and water. Throughout the experiment, all mice were maintained in a specific pathogen-free (SPF) state under a strict light cycle (on at 08:00 h; off at 20:00 h) at 23 ± 2 °C and 50 ± 10% relative humidity.

Animal models with UC symptoms were established with the treatment of either toxic chemicals (acetic acid, formalin, indomethacin, and TNBS) or immune reactive substances (DSS, carrageenan, immune complexes) [28,29,30]. DSS and male C57BL/6 mice were selected to successfully produce the UC model, as well as to rule out the excessive effects of hormones described in previous studies [20,22]. Briefly, these mice (*n* = 28) were assigned to either a non-UC group (No group, *n* = 7) or a UC group (DSS-treated group, *n* = 21). All mice in the UC groups received drinking water containing 2.5% DSS (MP biomedicals, California, USA) for 7 days. During this process, the UC group was further subdivided into one of three groups: Vehicle-treated group (DSS+Vehicle-treated group, *n* = 7), a group treated with a low concentration of AAS (DSS+LAAS-treated group, *n* = 7), and a group treated with a high concentration of AAS (DSS+HAAS-treated group, *n* = 7). The DSS+LAAS-treated group was orally administered 100 mg/kg body weight of AAS, while the DSS+HAAS-treated group received 200 mg/kg body weight of AAS at 10 a.m. for 7 days. The DSS+Vehicle-treated group was administered the same volume of dH_2_O in the same manner. In this period, animal survival and bloody stools were monitored at 10 a.m. every day. At 24 h after the final administration, all mice were euthanized using CO_2_ gas, after which colon, liver, and kidney samples were acquired and stored at −70 °C in Eppendorf tubes until assay.

### 2.5. Measurement of Body and Organ Weights

The body weights of the C57BL/6 mice in all subset groups were measured daily at 10:00 am for 7 days using an electronic balance (Mettler Toledo, Greifensee, Switzerland), according to the Korea Food and Drug Administration (KFDA) guidelines. In addition, the weights of the livers and kidneys from the euthanized mice were determined using the same electronic balance employed to measure body weight.

### 2.6. Disease Activity Index (DAI) Scoring System

DAI score was determined as described in previous studies [14,31]. During the experimental period, the body weight was measured daily, while the consistency and bleeding of stools were monitored every day. This score was classified into 13 stages, from 0 to 12, based on body weight loss, stool consistency, and gross bleeding. Each score was calculated as follows: body weight loss (0, none; 1, 1–5%; 2, 5–10%; 3, 11–15%; 4, >15%), stool consistency (0, normal; 2, loose stools; 4, watery diarrhea), and gross bleeding (0, normal; 2, hemoccult positive; 4, gross bleeding), and the individual scores were summed to obtain the aggregate score.

### 2.7. Measurement of Colon Length

After collection of the gastrointestinal (GI) tract from the C57BL/6 mice of each subset group, the colon region from the cecum to the anus was dissected from the GI tract. An image of their morphology was taken with a digital camera (Canon, Middlesex, UK). The total length of each colon was also measured in duplicate using a ruler.

### 2.8. Serum Biochemical Analysis

After the final administration, all C57BL/6 mice were fasted for 8 h, following which they were anesthetized with an intravenous injection of Alfaxan (JUROX Pty Limited, Rutherford, Australia, 13 mg/kg body weight i.v.). Whole blood was subsequently collected from the abdominal veins using a 1 mL syringe attached to a needle (26 SWG). The serum samples were obtained for biochemical analysis by centrifuging the whole blood at 1500× *g* for 15 min. Several serum biochemical components, including alkaline phosphatase (ALP), alanine aminotransferase (ALT), aspartate aminotransferase (AST), calcium (Ca), and low-density lipoprotein (LDH) were assayed using an automatic serum analyzer (Hitachi 747; Hitachi, Tokyo, Japan). All assays were measured in duplicate using a fresh serum.

### 2.9. Quantitative Real Time-PCR (RT-qPCR) Analysis

The total RNA was purified from the colon of the C57BL/6 mice using RNAzol (Tet-Test Inc., Friendswood, TX, USA). After determining the total RNA concentrations, complementary DNA (cDNA) was synthesized using Invitrogen Superscript II reverse transcriptase (Thermo Fisher Scientific Inc., Wilmington, DE, USA). The quantitative PCR was performed using the cDNA template (1 μL) and 2× Power SYBR Green (6 μL; Toyobo Life Science, Osaka, Japan) containing specific primers. The primer sequences used for the identification of target gene expression were as follows: TNF-α, sense 5′- CCT GTA GCC CAC GTC GTA -3′ and anti-sense, 5′- TTG ACC TCA GCG CTG ACT TG -3′, IL-1β, sense 5′- AGG CTT CCT TGT GCA AGT GT -3′ and anti-sense, 5′- TGA GTG ACA CTG CCT TCC TG -3′, IL-6, sense 5′- CTC TCT GCA AGA GAG TTC CAT CCA G -3′ and anti-sense, 5′- GCT ATG GTA CTC CAG AAG ACC AGA GG -3′, IL-10, sense 5′- CTC TTA CTG ACT GGC ATG AGG ATC AG -3′ and anti-sense, 5′- CTA TGC AGT TGA TGA AGA TGT CAA ATT C -3′, MUC2, sense 5′- GCA CAT TCC TTC GCA TCT TAA A -3′ and anti-sense, 5′- AAA GCA AAG AAT GGA ACA GAA CAG AAA CTC -3′. qPCR was performed over 40 cycles of denaturation at 95 °C for 15 s, annealing at 70 °C for 60 s, and extension at 70 °C for 60 s. Fluorescence intensities were measured at the end of the extension phase of each cycle. Threshold values for sample fluorescence intensities were set manually, and the reaction cycles in which PCR products exceeded the fluorescence intensity threshold during the exponential phase were considered to be the threshold cycles (Ct). Expressions of TNF-α, IL-1β, IL-6, IL-10, and MUC2 genes were quantified with respect to GAPDH (the housekeeping gene) by comparing Ct values at a constant fluorescence intensity, as described by Livak and Schmittgen [32].

### 2.10. Western Blot Analysis

The total protein content was obtained from the colon tissues of the C57BL/6 mice by applying Pro-Prep Protein Extraction Solution (Intron Biotechnology Inc., Seongnam, Republic of Korea) in accordance with the manufacturer’s protocol. After centrifugation at 13,000 rpm/min for 5 min, the protein concentrations were determined using a SMART™ Bicinchoninic Acid Protein Assay Kit (Thermo Fisher Scientific Inc.). Proteins were separated by 4–20% sodium dodecyl sulfate-polyacrylamide gel electrophoresis (SDS-PAGE) for 2 h and then transferred to nitrocellulose membranes at 40 V for 2 h. Membranes were then incubated at 4 °C with the following primary antibodies overnight: anti-p38 (Cell Signaling Technology Inc., Cambridge, MA, USA), anti-p-p38 (Cell Signaling Technology Inc.), anti-Protein kinase C (PKC) (Cell Signaling Technology Inc.), anti-phospho-PKC (p-PKC) (Cell Signaling Technology Inc.), anti- Akt serine/threonine kinase (AKT) (Cell Signaling Technology), anti-p-AKT (Cell Signaling Technology), anti-Extracellular signal-regulated kinase (ERK) (Cell Signaling Technology Inc.), anti-p-ERK (Cell Signaling Technology Inc.), anti-PI3K (Cell Signaling Technology), anti-p-PI3K (Cell Signaling Technology), anti- c-Jun N-terminal kinases (JNK) (Cell Signaling Technology Inc.), anti-p-JNK (Cell Signaling Technology Inc.), or anti- Glyceraldehyde 3-phosphate dehydrogenase (GAPDH) antibody (Cell Signaling Technology Inc.). The membranes were then washed with washing buffer (137 mM NaCl, 2.7 mM KCl, 10 mM Na_2_HPO_4_, and 0.05% Tween 20) and incubated with 1:2000 diluted horseradish peroxidase (HRP)-conjugated goat anti-rabbit IgG (Invitrogen) at room temperature for 1 h. Blots were developed using Amersham ECL Select Western Blotting detection reagent (GE Healthcare, Little Chalfont, UK). Chemiluminescence signals from specific bands were detected using FluorChemi^®^FC2 (Alpha Innotech Co., San Leandro, CA, USA).

### 2.11. Histopathological Analysis

Colons, livers, and kidneys collected from the No, DSS+Vehicle, DSS+LAAS, and DSS+HAAS-treated C57BL/6 mice were fixed in 10% formalin for 48 h. Samples were subsequently embedded in paraffin wax, after which they were cut into 4 μm thick sections and stained with hematoxylin and eosin (H&E, Sigma-Aldrich Co.). Alterations to the histopathological structure of the stained sections were then analyzed using light microscopy, applying the Leica Application Suite (Leica Microsystems, Glattbrugg, Switzerland). Based on the above results, (1) the severity of inflammation (0, none; 1, mild; 2, moderate; 3, severe); (2) the extent of inflammation (0, none; 1, mucosa; 2, mucosa and submucosa; 3, transmural); and (3) crypt damage (0, none; 1, 1/3 damaged; 2, 2/3 damaged; 3, crypt loss but surface epithelium present; 4, both crypt and surface epithelium lost) were determined. Finally, the histological score was calculated as described in a previous study [33]. All pathological features were characterized by a pathologist, Prof. Beum Seok Han, at the Hoseo University (Asana, Republic of Korea).

Mucin staining was achieved by fixing the colons collected from the C57BL/6 mice of all subset groups in 10% formalin for 48 h, then embedding the samples in paraffin wax and sectioning into 4 μm thick slices that were subsequently deparaffinized with xylene and rehydrated. The mounted tissue sections were rinsed with distilled water and stained using an Alcian Blue Stain kit (Ihc World LLC, Woodstock, MD, USA), and the morphological features in the stained colon sections were observed by light microscopy. Furthermore, the histopathological analyses including slide preparation, H&E staining, mucin staining, and microscopic observation, were conducted randomly.

### 2.12. Statistical Significance Analysis

Statistical analyses were performed using SPSS release 27 for Windows (IBM SPSS, SPSS Inc., Chicago, IL, USA). The significance of intergroup differences was determined using one-way analysis of variance followed by Tukey’s post hoc test for multiple comparisons. Results are presented as means ± SDs, and statistical significance was accepted for *p* values < 0.05.

## 3. Results

### 3.1. Active Components and Antioxidant Activity of AAS

The concentration of active components of AAS was assessed using the HPLC method and the antioxidant activity in AAS was measured with a DPPH scavenging assay. Total polyphenol content and condensed tannin contents were 272 ± 13 μg GAE/g and 163 ± 16 μg CHE/g, respectively (Figure 1b). In addition, inhibitory activity against DPPH radicals was significantly increased in a dose-dependent manner at 1, 2, and 4 mg/mL of AAS (Figure 1c).

### 3.2. Treatment with AAS Improves Clinical Symptoms of UC in DSS-Induced UC Mice

**Figure 2:**
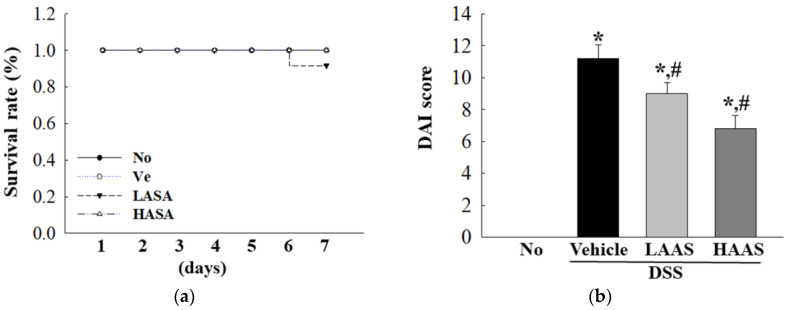

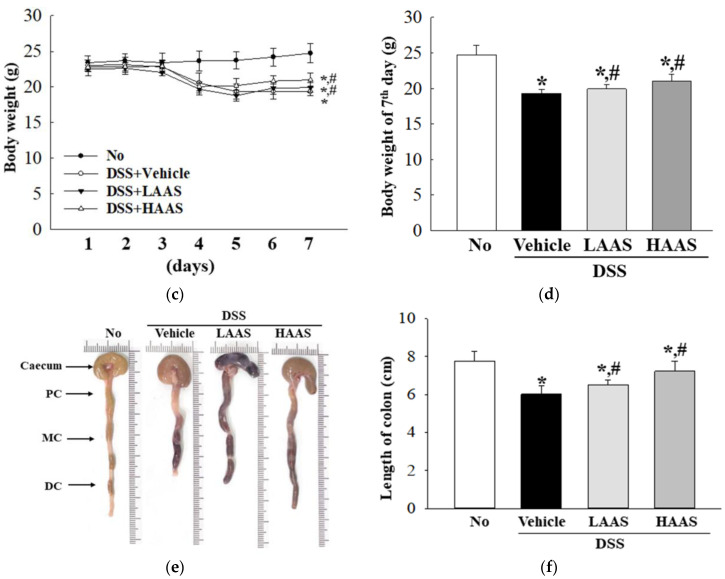
Clinical symptoms in DSS+AAS-treated mice. (**a**) Survival rate during the 7 days of treatment. Survival rate was monitored in all mice of the subset groups, as described in Materials and Methods section. (**b**) DAI score. This score was calculated by combining scores of three factors including body weight loss, stool consistency, and gross bleeding. (**c**) Changes in body weight during the 7 days of treatment. (**d**) Body weight at 7th day. The body weight of No, DSS+Vehicle, DSS+LAAS and DSS+HAAS-treated groups were measured from 0 to 7 days weeks, using a chemical balance. (**e**) Actual image of colon. After collection of colons, the total length from caecum to anus was measured using a ruler. Their morphology was observed using a digital camera. (**f**) Length of colons. This value is represented as a bar graph and statistical significance is indicated. Seven mice per group were used to prepare the clinical symptom analysis, and survival rate, DAI score, body weight and colon length analysis were measured in duplicate. The data are reported as the mean ± SD. *, *p* < 0.05 relative to the No group. ^#^, *p* < 0.05 relative to the DSS+Vehicle-treated group. Abbreviations: AAS, aqueous extract of *A. saponaria*; DSS, Dextran sodium sulfate; DAI, Disease activity index; No: Non-UC group; LAAS: Low dose of AAS; HAAS: High dose of AAS; PC, Proximal colon; MC, Mid colon; DC, Distal colon.

To investigate whether the administration of AAS could improve the clinical symptoms of UC, the survival rate, DAI score, body weight, hematochezia, and colon length were measured in the DSS-induced UC C57BL/6 mice. First, the survival rate was maintained in all experimental groups (Figure 2a). The DAI score remarkably decreased in the DSS+LAAS and DSS+HAAS-treated groups compared to the DSS+Vehicle-treated group (Figure 2b). In addition, the body weight was lower in the DSS+Vehicle-treated group than in the No group from the 4th to the 7th day. However, body weight significantly increased in the DSS+LAAS and DSS+HAAS-treated groups compared to the DSS+Vehicle-treated group on the 7th day (Figure 2c,d). The DSS+LAAS and DSS+HAAS-treated groups showed improvements in colon length of about 10% and 21%, respectively, compared to the DSS+Vehicle-treated group (Figure 2e,f).

### 3.3. Treatment with AAS Leads to Improvement in the Morphological and Histopathological Characteristics of the Colon in DSS-Induced UC Mice

**Figure 3:**
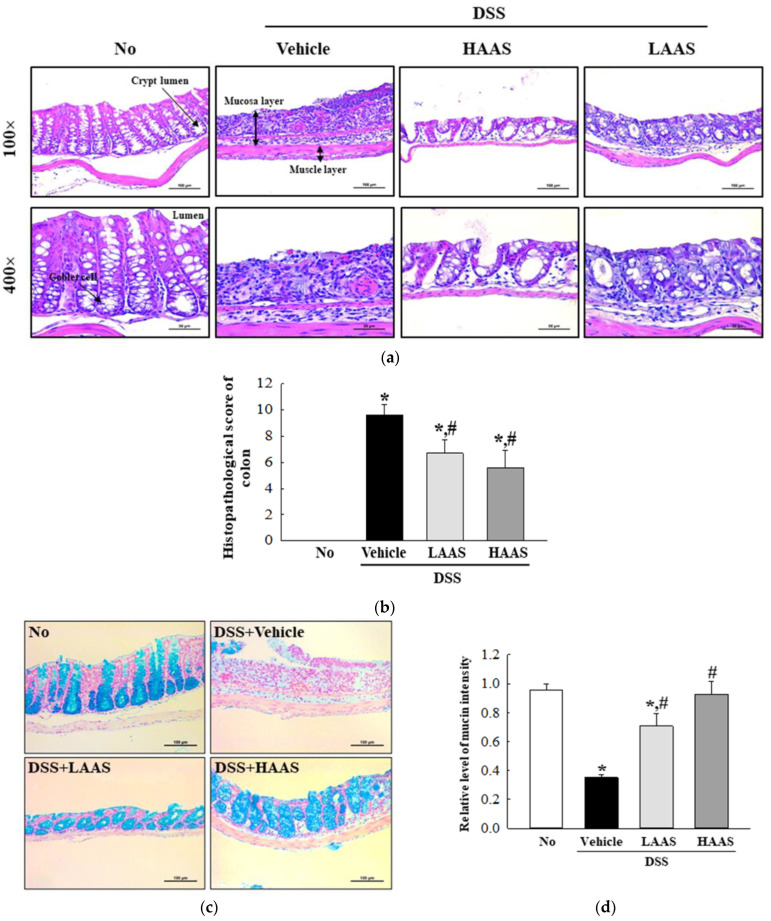

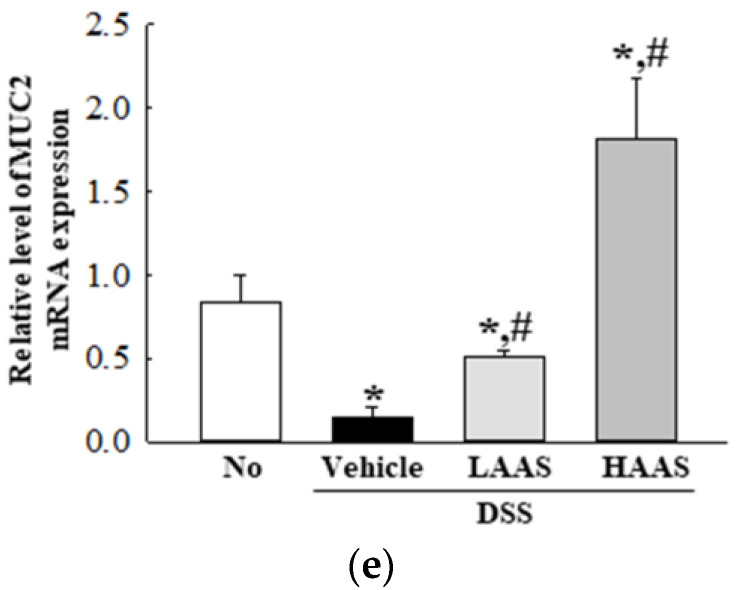
Histopathological characteristics and mucin secretion in the colon of DSS+AAS-treated mice. (**a**) Histological structure. Colon tissues were stained with H&E and cellular morphology was viewed at 100× and 400× magnification. (**b**) Histopathological score. This was determined based on the severity of inflammation, the extent of inflammation, and crypt damage. (**c**,**d**) Mucin staining analyses. Mucin secreted from the crypt layer cells was stained with Alcian blue at pH 2.5, and images were observed at 100× magnification. Three to five mice per group were used to prepare Alcian blue stained slides, and staining density was analyzed in duplicate for each slide. (**e**) Expression of MUC2. The levels of MUC2 transcripts in the total mRNA of mid colons were measured by RT-qPCR using specific primers. The mRNA level of this gene was calculated, based on the intensity of GAPDH as an endogenous control. Five to seven mice per group were used to prepare Histopathological characteristics and mucin secretion analyses, and H&E staining, mucin staining, and RT-qPCR was analyzed in duplicate for each sample. The data are reported as the mean ± SD. *, *p* < 0.05 relative to the No group. ^#^, *p* < 0.05 relative to the DSS+Vehicle-treated group. Abbreviation: Abbreviation: AAS, aqueous extract of *A. saponaria*; DSS, Dextran sodium sulfate; No: Non-UC group; LAAS: Low dose of AAS; HAAS: High dose of AAS; H&E stain: hematoxylin and eosin stain; MUC2, Mucin 2; RT-qPCR: Quantitative Real Time-PCR.

To investigate whether the administration of AAS could improve the histopathological characteristics, changes in the histological scores were analyzed in the H&E-stained colons of DSS-induced UC mice. The DAI scores, which included the severity and extent of inflammation, and crypt damages were high in the DSS-induced UC mice group when compared to the No group. These scores were remarkably improved in the DSS+LAAS and DSS+HAAS-treated groups (Figure 3a,b). In addition, a similar recovery was observed in the mucin secretion ability. The dark blue stained intensity of mucin in the goblet cells of the crypt disappeared in the colon of the DSS+Vehicle-treated group compared to the No group. The mucin-secreting capacity of the goblet cells significantly improved after the AAS treatment, although this was not completely restored in the No group (Figure 3c,d).

### 3.4. Treatment with AAS Attenuated the Inflammatory Response in the Colon of DSS-Induced UC Mice

**Figure 4:**
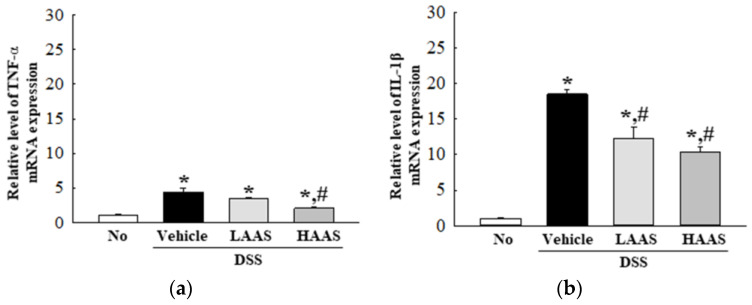

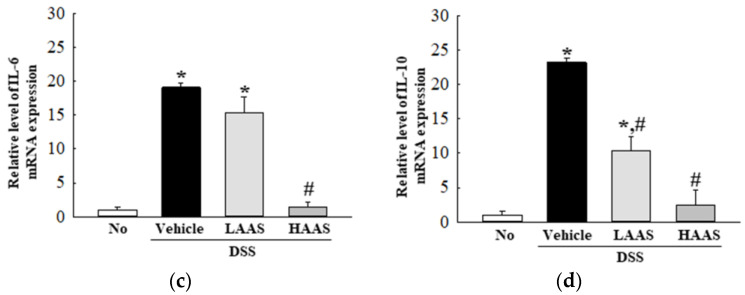
Expression level of pro-inflammatory cytokines in DSS-induced UC model after administration of AAS. The levels of TNF-α (**a**), IL-1β (**b**), IL-6 (**c**), and IL-10 (**d**) transcripts were detected in the total mRNA of colon tissue by performing qRT-PCR with specific primers. Five to seven mice per group were used to prepare the total RNAs, and qRT-PCR was performed in duplicate for each sample. Data are reported as the mean ± SD. *, *p* < 0.05 relative to the No group. ^#^, *p* < 0.05 relative to the DSS+Vehicle-treated group. Abbreviation: AAS, aqueous extract of *A. saponaria*; RT-qPCR, quantitative real time-PCR; TNF-α, Tumor necrosis factor-alpha; IL, Interleukin.

To investigate whether the administration of AAS improved the inflammatory response, changes in the infiltration of inflammatory cells and cytokine expression were measured in the colons of DSS-induced UC mice. The number of infiltrated cells was significantly higher in the DSS+Vehicle-treated group than in the No group. However, these numbers decreased in the DSS+LAAS and DSS+HAAS-treated groups compared to the DSS+Vehicle-treated group (Figure 3a). Moreover, the expression of colonic pro-inflammatory mediators including TNF-α, IL-1β, IL-6, and IL-10 was assessed. In the DSS+LAAS and DSS+HAAS-treated groups, the expression of the pro-inflammatory mediators remarkably decreased in a dose-dependent manner compared to the DSS+Vehicle-treated group. Specifically, a significant decrease was detected in the IL-6 and IL-10 expressions in the DSS+HAAS-treated group (Figure 4).

### 3.5. Effects of AAS on the PI3K/Akt and PKC/ERK Signaling Pathway in the Colon of DSS-Induced UC Mice

**Figure 5:**
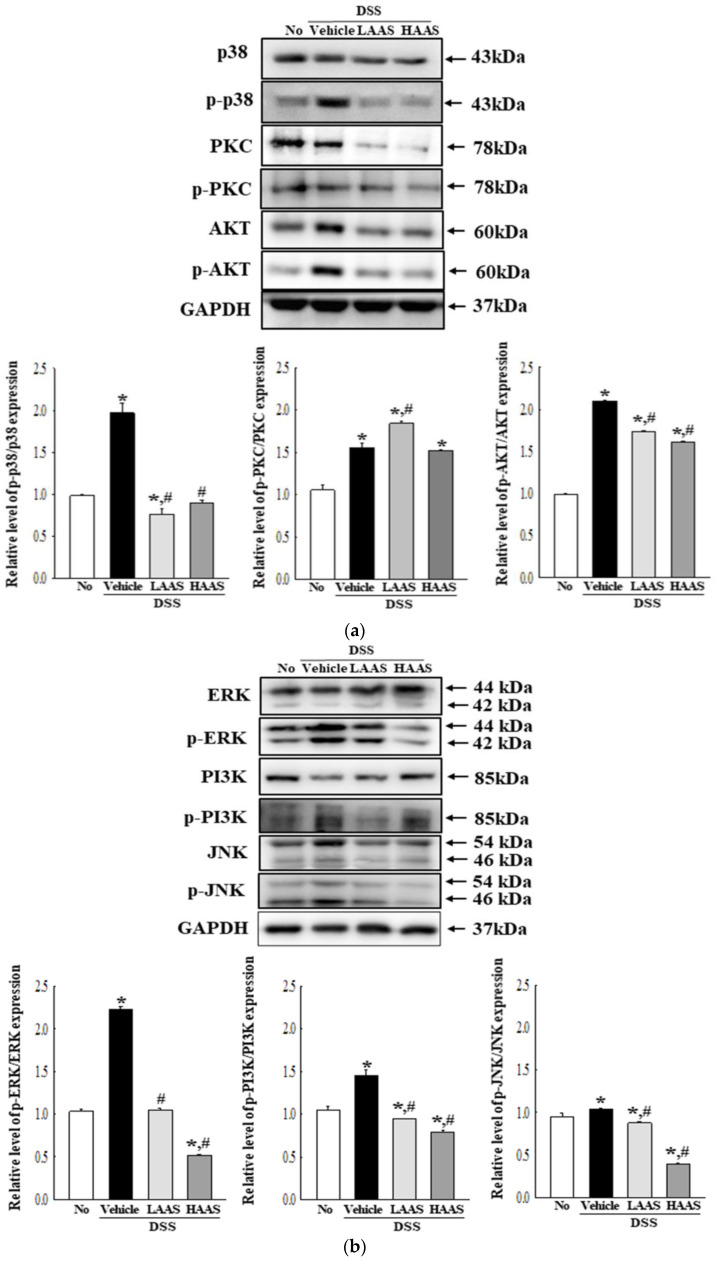
PI3K/Akt signaling pathway and PKC/ERK signaling pathway in DSS+AAS-treated mice. (**a**) Level of key members in the PI3K/Akt signaling pathways. Expression levels of six proteins were determined using an imaging densitometer. The level of each protein is presented relative to the intensity of GAPDH. (**b**) Level of key members in PKC/ERK signaling pathway. Expression levels of six proteins were determined using an imaging densitometer. The level of each protein is presented relative to the intensity of GAPDH. Five to seven mice per group were used to prepare tissue homogenates, and Western blot analysis was performed in duplicate for each sample. Data are reported as the mean ± SD. *, *p* < 0.05 relative to the No group. ^#^, *p* < 0.05 relative to the DSS+Vehicle-treated group. Abbreviation: AAS, aqueous extract of *A. saponaria*; PKC, Protein kinase C; Akt, Serine-threonine protein kinase; ERK, Extracellular signal regulated kinase; PI3K, Phosphoinositide 3-kinase; JNK, c-Jun N-terminal kinase.

Next, we determined whether the anti-inflammatory effects of AAS were accompanied by regulation of the PI3K/Akt and PKC/ERK signaling pathways in the colon of the UC mice. To achieve this, alterations in the expression of key regulator proteins were measured in the colon of DSS-induced UC mice. In the PI3K/Akt signaling pathway, the phosphorylation of p38, PKC, and Akt was higher in the DSS+Vehicle-treated group than the No group. However, phosphorylation significantly decreased after the AAS treatment, although the level of PKC was maintained (Figure 5a). A similar decrease was observed in the PKC/ERK signaling pathway. The phosphorylation of ERK, PI3K, and JNK decreased in a dose-dependent manner in the DSS+AAS-treated group, although this was higher in the DSS+Vehicle-treated group compared to the No group (Figure 5b).

### 3.6. Hepatotoxicity and Nephrotoxicity of AAS

**Figure 6:**
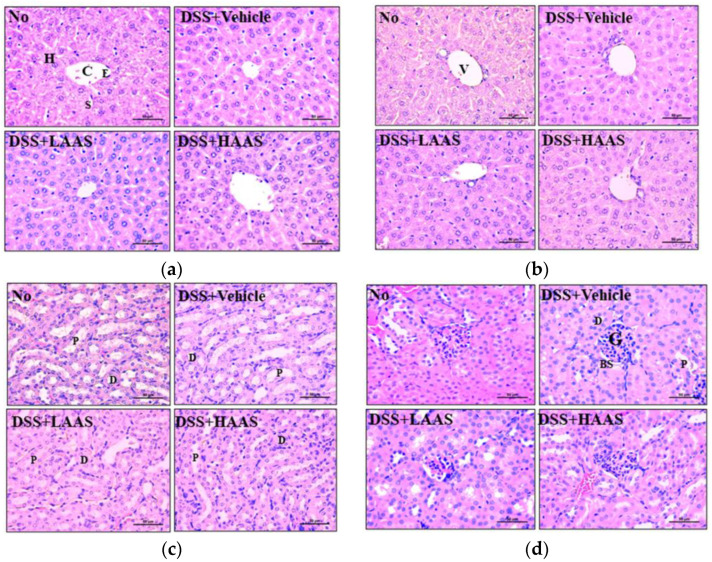
Histopathological features of the liver and kidney in DSS+AAS-treated mice. (**a**) Structure around the pericentral region in liver. (**b**) Structure around of the periportal region in liver. (**c**) Tubule region in kidney. (**d**) Glomerulus region in kidney. Liver and kidney tissues were stained with H&E and cellular morphology was viewed at 400× magnification. Five to seven mice per group per group were used to prepare H&E stained tissue slides, and histopathological alterations were analyzed in duplicate for each sample. Abbreviation: AAS, aqueous extract of *A. saponaria*; C, Central vein; H, Hepatocytes; E, Endothelial cells; V, Portal vein.; H&E stain: hematoxylin and eosin stain; G, Glomeruli; BS, Bowman’s space; P, proximal convoluted tubule; D, distal convoluted tubule.

Finally, we investigated whether the administration of AAS for 7 days caused hepatotoxicity and nephrotoxicity in C57BL/6 mice. For this purpose, changes in the organ weight, and serum biochemical profile, and histopathological changes in the livers and kidneys of the C57BL/6 mice administered AAS for 7 days were analyzed. No significant differences were detected in the liver and kidney weight between the mice of all subset groups (Table 1). Similarly, the serum concentration of liver enzymes including ALP, AST, and ALP as well as biomarkers of kidney function including BUN and serum creatinine did not change between the groups (Table 1). Furthermore, there were no significant histopathological changes, including inflammation, necrosis, apoptosis, and fibrosis, in the H&E-stained liver sections of the DSS+AAS-treated mice (Figure 6a,b). Moreover, no nephrotoxicity, including degeneration and necrosis of the glomerulus and renal tubes was detected in the histopathological analysis of the kidneys of the DSS+LAAS and DSS+LAAS-treated groups (Figure 6c,d).

## 4. Discussion

To date, the drugs available for the treatment of UC and other forms of IBD are limited in their efficacy and tolerability [11]. Therefore, there is a constant need for the development of effective novel drugs. In addition, the current incidence of UC is at 200–250 per 100,000, and this rate is increasing worldwide [34]. As part of this study, we evaluated whether *A. saponaria* has therapeutic potential as a treatment for UC. To achieve this, changes in the clinical symptoms, histopathological characteristics, and inflammatory response in a DSS-induced UC model were analyzed after the administration of AAS for 7 days. The results indicate that AAS can improve the symptoms of UC by enhancing the function of the mucus barrier and suppressing the inflammatory responses in the colon, although more studies are needed to elucidate the mechanism of action of the bioactive components present in AAS.

Several natural products have been investigated at various concentrations to evaluate their therapeutic effectiveness and the molecular mechanism in the treatment of UC. The aqueous extract of *P. san* effectively relieved the symptoms of UC in a DSS-induced UC model at relatively high concentrations of 2, 4, and 8 g/kg [14]. Similar therapeutic effects against UC were detected with low concentrations (200, 400, and 800 mg/kg) of the aqueous extract of *H. littoralis* [13]. The ethanol extracts of *A. vera* at 200 and 400 mg/kg improved the symptoms of UC in TNBS-induced UC mice, while significant improvements in the DAI score, colon length, histopathological characteristics, inflammation, and mucin secretion were detected at low doses (18 and 72 mg/kg) of *A. vera* aqueous extracts [21,22]. Furthermore, antioxidant formulations of *A. vera* and ubiquinol exhibited protective and healing effects against DSS-induced UC at 5 and 25 mg/kg [20]. In the present study, AAS was orally administrated to a DSS-induced UC model at 100 and 200 mg/kg concentrations, to evaluate the anti-UC effects. These concentrations of AAS significantly improved the symptoms of UC. The concentrations applied in the present study fall within the range of concentrations of extracts used for treatment in previous studies, although those in our study belong to the lower end of the range.

Weight loss, bloody stools, and shortened colon lengths are well-known clinical symptoms of UC [35]. A significant loss of body weight was induced by the decrease of muscle mass, by enhancing the breakdown of muscle and fat-free area mass, and large amounts of total proteins were thus required due to chronic inflammation [36]. In addition, decrease in colon length is caused by the atrophy of the colon during ulceration, mucosal bleeding, and edema from the rectum to the colon [37]. Bloody stools are associated with small ulcerations at various sites in the rectum and colon as well as a decrease in mucus secretion in some individuals with severe UC [38]. The above symptoms of UC improved significantly in UC models after treatment with several natural products. Administration of *P. san* and *H. littoralis* extracts resulted in remarkable recovery, with body weight gain and lengthening of the colon length in DSS-induced UC mice [13,14]. Furthermore, similar improvements in the clinical symptoms were detected in a TNBS-induced UC model treated with ethanol extracts of *A. vera* leaves and a DSS-induced UC model treated with aqueous extracts of *A. vera* [21,22]. In the present study, the improvement in symptoms of UC was assessed in DSS-induced UC mice after the administration of AAS. The loss of body weight, shortening of colon length, and increase of bloody stools improved with the administration of AAS. Our results, with improvements in the clinical symptoms of the AAS-treated UC models, were very similar to those from previous studies with several other natural products, although the treated extracts and the method for producing the UC model were different.

Disruption of the colon, including a decrease in crypt density, architectural distortion of the crypt, irregular mucosal surface, and diffusion of transmucosal inflammation, as seen in histopathological studies, are considered major markers of the severity of UC [5,6]. Based on this scientific evidence, our study analyzed alterations in the histopathological characteristics of the colon in DSS-induced UC mice, to provide additional scientific evidence for the effectiveness of a novel natural product, AAS. As shown in Figure 6, the loss of epithelial cells, disruption of the crypt structure, and thickness of the crypt on the H&E-stained colon section recovered in the DSS-induced UC model after treatment with AAS for 7 days. Similar alterations in the histopathological structure were detected in previous studies that investigated the effects of several natural products in UC. Mucosal damage, loss of goblet cells, crypt damage, and infiltration of immune cells improved in DSS-induced UC mice after treatment with *P. san*, while the histopathological scores reflecting the structural alterations in the colon also improved in DSS-induced UC mice after administration of *H. littoralis* [13,14]. Administration of *A. vera* gel for 21 days reduced inflammation, ulcers, and tissue damage in the colon, as seen with H&E staining in mice with acetic acid-induced UC [16]. In a TNBS-induced UC model, histological changes, including cellular damage, infiltration of immune cells, edema of the colon mucosa, and distortion of the crypt architecture, were attenuated and improved when treated with *A. vera* extracts [21]. Furthermore, similar improvements were detected in the H&E-stained distal colon sections of DSS-induced UC mice after treatment with *A. vera* extract for 10 days [22].

Cytokines play a key role in the immune response of the gastrointestinal (GI) tract through the facilitation of communication between cells, stimulation of the proliferation of effector cells, and mediation of local and systemic inflammation [39,40]. During the pathogenesis of IBD, including UC and Crohn’s Disease (CD), various cytokines such as tumor necrosis factor-alpha (TNF-α), interferon-gamma (INF-γ), interleukin (IL)-1, IL-6, IL-4, IL-5, IL-10, tumor growth factor-beta (TGF-β) are secreted from activated macrophages and dendritic cells to control the inflammatory response [41,42]. The concentration of these cytokines is upregulated or overactivated, subsequently leading to T cell dysregulation and an imbalance of Treg/Th1, Th2, and Th17 cells [42,43]. Various therapeutic drugs and natural products used for the treatment of UC can effectively improve the dysregulation in the concentration and expression levels of these cytokines. Ethanol and aqueous extracts of *A. vera* suppressed colonic pro-inflammatory mediators, including IL-6, IL-1β, and TNF-α in TNBS- and DSS-induced UC models, although they did not affect the levels of IL-10 [21,22]. Furthermore, similar suppression was seen in a UC model after treatment with *P. san* and *H. littoralis* extracts. Treatment with *P. san* in DSS-induced UC mice induced a dose-dependent suppression of the expression of TNF-α, IL-1β, and IL-12 mRNA, while a *H. littoralis* treatment showed a steady decrease in the concentration of TNF-α, IFN-γ, IL-6, and IL-1β proteins in the colorectal tissue homogenate of the same model [13,14]. In the present study, we measured the expression levels of TNF- α, IL-1β, IL-6, and IL-10 mRNA, to verify the effects of AAS treatment on the inflammatory response. These levels were remarkably decreased after the AAS treatment. The above results from the AAS-treated UC model were very similar to those of previous studies, although there were differences in the type and inhibition rate of cytokines.

## 5. Conclusions

In the present study, we attempted to demonstrate the novel therapeutic effects of AAS in UC using DSS-treated C57BL/6 mice. For this purpose, the present study investigated the biochemical properties of AAS, as well as alterations in clinical symptoms, histopathological characteristics, and anti-inflammatory response in a DSS-induced UC model after the administration of AAS for 7 days. In addition, hepatotoxicity and nephrotoxicity were further evaluated in the same model, to evaluate its safety. Our results provide the first scientific evidence that AAS has great potential to relieve the symptoms of UC, although further research will be needed to elucidate the mechanisms of action of the bioactive components of AAS.

## Figures and Tables

**Table 1:** Organ weights and the level of serum biochemical factors in DSS-induced UC mice model after administration of AAS.

Category		No	DSS			Statistical Difference
			Vehicle	LAAS	HAAS	
Organ weights	Liver (g)	1.39 ± 0.10	0.97 ± 0.03	0.85 ± 0.04	0.89 ± 0.06	No significance
	Kidney (g)	0.36 ± 0.03	0.32 ± 0.02	0.32 ± 0.02	0.32 ± 0.04	No significance
Serum biochemical factor	ALT (U/L)	30.4 ± 2.8	31.5 ± 8.1	31.75 ± 8.6	34.0 ± 5.2	No significance
	AST (U/L)	47.0 ± 3.3	44.3 ± 4.0	48.0 ± 1.7	46.75 ± 1.1	No significance
	ALP (g/mL)	91.0 ± 2.8	90.0 ± 4.2	88.0 ± 7.0	88.5 ± 2.1	No significance
	BUN (mg/dL)	23.8 ± 4.0	26.9 ± 2.2	28.8 ± 4.1	29.7 ± 4.0	No significance
	Crea (mg/dL)	0 ± 0	0 ± 0	0 ± 0	0 ± 0	No significance

## Data Availability

Data is contained within the article.

## References

[1] Baumgart D.C., Sandborn W.J. (2007). Inflammatory bowel disease: Clinical aspects and established and evolving therapies. Lancet.

[2] Gajendran M., Loganathan P., Jimenez G., Catinella A.P., Ng N., Umapathy C., Ziade N., Hashash J.G. (2019). A comprehensive review and update on ulcerative colitis. Dis. A Mon..

[3] Lombardi V.R., Etcheverría I., Carrera I., Cacabelos R., Chacón A.R. (2012). Prevention of chronic experimental colitis induced by dextran sulphate sodium (DSS) in mice treated with FR91. J. Biomed. Biotechnol..

[4] Turner D., Levine A., Escher J.C., Griffiths A.M., Russell R.K., Dignass A., Dias J.A., Bronsky J., Braegger C.P., Cucchiara S. (2012). Management of pediatric ulcerative colitis: Joint ECCO and ESPGHAN evidence-based consensus guidelines. J. Pediatr. Gastroenterol. Nutr..

[5] Bentley E., Jenkins D., Campbell F., Warren B. (2002). How could pathologists improve the initial diagnosis of colitis? Evidence from an international workshop. J. Clin. Pathol..

[6] Seldenrijk C.A., Morson B.C., Meuwissen S.G., Schipper N.W., Lindeman J., Meijer C.J. (1991). Histopathological evaluation of colonic mucosal biopsy specimens in chronic inflammatory bowel disease: Diagnostic implications. Gut.

[7] Lu A., Magupalli V.G., Ruan J., Yin Q., Atianand M.K., Vos M., Schröder G.F., Fitzgerald K.A., Wu H., Egelman E.H. (2014). Unified polymerization mechanism for the assembly of ASC-dependent inflammasomes. Cell.

[8] Rahimian R., Zirak M.R., Keshavarz M., Fakhraei N., Mohammadi-Farani A., Hamdi H., Mousavizadeh K. (2016). Involvement of PPAR γ in the protective action of tropisetron in an experimental model of ulcerative colitis. Immunopharmacol. Immunotoxicol..

[9] Yadav P.N., Liu Z., Rafi M.M. (2003). A diarylheptanoid from lesser galangal (*Alpinia officinarum*) inhibits proinflammatory mediators via inhibition of mitogen-activated protein kinase, p44/42, and transcription factor nuclear factor-κB. J. Pharmacol. Exp. Ther..

[10] Kaliyeva S., Simohina N., Yukhnevich Y., Myasnikova Z., Myasnikov Y. (2018). Pharmacoeconomic assessment of biological therapy of ulcerative colitis. Value Health.

[11] Gupta M., Mishra V., Gulati M., Kapoor B., Kaur A., Gupta R., Tambuwala M.M. (2022). Natural compounds as safe therapeutic options for ulcerative colitis. Inflammopharmacology.

[12] Zhang S.Z., Zhao X.H., Zhang D.C. (2006). Cellular and molecular immunopathogenesis of ulcerative colitis. Cell. Mol. Immunol..

[13] Lin G., Li M., Xu N., Wu X., Liu J., Wu Y., Zhang Q., Cai J., Gao C., Su Z. (2020). Anti-inflammatory effects of *Heritiera littoralis* fruits on dextran sulfate sodium-(DSS-) induced ulcerative colitis in mice by regulating gut microbiota and suppressing NF-κB pathway. BioMed Res. Int..

[14] Zhang Z., Shen P., Xie W., Cao H., Liu J., Cao Y., Zhang N. (2019). *Pingwei San* ameliorates dextran sulfate sodium-induced chronic colitis in mice. J. Ethnopharmacol..

[15] Babalola W.O., Ofusori D.A., Awoniran P., Falana B.A. (2022). *Aloe vera* gel attenuates acetic acid-induced ulcerative colitis in adult male Wistar rats. Toxicol. Rep..

[16] Bahrami G., Malekshahi H., Miraghaee S., Madani H., Babaei A., Mohammadi B., Hatami R. (2020). Protective and therapeutic effects of *Aloe vera* gel on ulcerative colitis induced by acetic acid in rats. Clin. Nutr. Res..

[17] Hassanshahi N., Masoumi S.J., Mehrabani D., Hashemi S.S., Zare M. (2020). The healing effect of *Aloe vera* gel on acetic acid-induced ulcerative colitis in rat. Middle East J. Dig. Dis..

[18] Langmead L., Feakins R.M., Goldthorpe S., Holt H., Tsironi E., De Silva A., Jewell D.P., Rampton D.S. (2004). Randomized, double-blind, placebo-controlled trial of oral *Aloe vera* gel for active ulcerative colitis. Aliment. Pharmacol. Ther..

[19] Radha M.H., Laxmipriya N.P. (2015). Evaluation of biological properties and clinical effectiveness of *Aloe vera*: A systematic review. J. Tradit. Complement. Med..

[20] Korkina L., Suprun M., Petrova A., Mikhal’Chik E., Luci A., Luca C.D. (2003). The protective and healing effects of a natural antioxidant formulation based on ubiquinol and *Aloe vera* against dextran sulfate-induced ulcerative colitis in rats. Biofactors.

[21] Naini M.A., Zargari-Samadnejad A., Mehrvarz S., Tanideh R., Ghorbani M., Dehghanian A., Hasanzarrini M., Banaee F., Hosseinabadi O.K., Iraji A. (2021). Anti-inflammatory, antioxidant, and healing-promoting effects of *Aloe vera* extract in the experimental colitis in rats. Evid. Based. Complement. Altern. Med..

[22] Choi S.M., Supeno D., Byun J.Y., Kwon S.H., Chung S.W., Kwon S.G., Park J.M., Kim J.S., Kwon D.Y., Choi W.S. (2016). Chemical characteristics of *Aloe vera* and *Aloe saponaria* in Ulsan Korea. Int. J. Bio-Sci. Bio-Technol..

[23] Shi G., Jiang H., Feng J., Zheng X., Zhang D., Jiang C., Zhang J. (2021). *Aloe vera* mitigates dextran sulfate sodium-induced rat ulcerative colitis by potentiating colon mucus barrier. J. Ethnopharmacol..

[24] Kumaran A., Karunakaran R.J. (2007). In vitro antioxidant activities of methanol extracts of five *Phyllanthus* species from India. LWT-Food Sci. Technol..

[25] Price M.L., Hagerman A.E., Butler L.G. (1980). Tannin content of cowpeas, chickpeas, pigeon peas, and mung beans. J. Food Chem..

[26] Hassan S.M., Al Aqil A.A., Attimarad M. (2013). Determination of crude saponin and total flavonoids content in guar meal. Adv. Med. Plant Res..

[27] Lee S.J., Kim J.E., Choi Y.J., Gong J.E., Park S.H., Douangdeuane B., Souliya O., Park J.M., Lee H.S., Kim B.H. (2021). Therapeutic effects of *Dipterocarpus tuberculatus* with high antioxidative activity against UV-induced photoaging of NHDF cells and nude mice. Antioxidants.

[28] Kitajima S., Takuma S., Morimoto M. (2000). Histological analysis of murine colitis induced by dextran sulfate sodium of different molecular weights. Exp. Anim..

[29] Xia B., Deng C.S., Chen D.J., Zhou Y., Xiao J.Q. (1996). Role of copper zinc superoxide dismutase in the short-term treatment of acetic acid-induced colitis in rats. Acta Gastroenterol. Latinoam..

[30] Zea-Iriarte W.L., Makiyama K., Goto S., Murase K., Urata Y., Sekine I., Hara K., Kondo T. (1996). Impairment of antioxidants in colonic epithelial cells isolated from trinitrobenzene sulphonic acid-induced colitis rats protective effect of rebamipide. Scand. J. Gastroenterol..

[31] Kihara N., de la Fuente S.G., Fujino K., Takahashi T., Pappas T.N., Mantyh C.R. (2003). Vanilloid receptor-1 containing primary sensory neurons mediate dextran sulphate sodium induced colitis in rats. Gut.

[32] Livak K.J., Schmittgen T.D. (2001). Analysis of relative gene expression data using real-time quantitative PCR and the 2− ΔΔCT method. Methods.

[33] Andujar I., Recio M.C., Giner R.M., Cienfuegos-Jovellanos E., Laghi S., Muguerza B., Rios J.L. (2011). Inhibition of ulcerative colitis in mice after oral administration of a polyphenol-enriched cocoa extract is mediated by the inhibition of STAT1 and STAT3 phosphorylation in colon cells. Food Chem..

[34] Porter R.J., Kalla R., Ho G.T. (2020). Ulcerative colitis: Recent advances in the understanding of disease pathogenesis. F1000Research.

[35] Sninsky C.A. (2010). New research in ulcerative colitis: Optimizing 5-ASA administration for efficacy and adherence. Gastroenterol. Hepatol..

[36] Lee D., Albenberg L., Compher C., Baldassano R., Piccoli D., Lewis J.D., Wu G.D. (2015). Diet in the pathogenesis and treatment of inflammatory bowel diseases. Gastroenterology.

[37] Peyrin-Biroulet L., Desreumaux P., Sandborn W.J., Colombel J.F. (2008). Crohn’s disease: Beyond antagonists of tumor necrosis factor. Lancet.

[38] Adams S.M., Close E.D., Shreenath A.P. (2022). Ulcerative colitis: Rapid evidence review. Am. Fam. Physician.

[39] Jump R.L., Levine A.D. (2004). Mechanisms of natural tolerance in the intestine: Implications for inflammatory bowel disease. Inflamm. Bowel Dis..

[40] Neuman M.G. (2007). Immune dysfunction in inflammatory bowel disease. Transl. Res..

[41] Leon F., Smythies L.E., Smith P.D., Kelsall B.L. (2006). Involvement of dendritic cells in the pathogenesis of inflammatory bowel disease. Adv. Exp. Med. Biol..

[42] Papadakis K.A., Targan S.R. (2000). Role of cytokines in the pathogenesis of inflammatory bowel disease. Annu. Rev. Med..

[43] Sanchez-Muñoz F., Dominguez-Lopez A., Yamamoto-Furusho J.K. (2008). Role of cytokines in inflammatory bowel disease. World J. Gastroenterol..

